# Cardiogenic shock in phaeochromocytoma multisystem crisis: a case report

**DOI:** 10.1093/ehjcr/ytae463

**Published:** 2024-09-09

**Authors:** Yun Yun Go, Audrey Jing Ting Ng, Iswaree Devi Balakrishnan, Raj Vikesh Tiwari, Aaron Kian Ti Tong, Lianne Ai Ling Lee, Yann Shan Keh, Donovan Tay

**Affiliations:** Department of Cardiology, National Heart Centre Singapore, 5 Hospital Dr, Singapore 169609, Singapore; Department of Urology, Sengkang General Hospital, 110 Sengkang E Wy, Singapore 544886, Singapore; Department of Cardiology, National Heart Centre Singapore, 5 Hospital Dr, Singapore 169609, Singapore; Department of Urology, Sengkang General Hospital, 110 Sengkang E Wy, Singapore 544886, Singapore; Department of Nuclear Medicine and Molecular Imaging, Singapore General Hospital, 110 Sengkang E Wy, Singapore 544886, Singapore; Department of Pathology, Sengkang General Hospital, 110 Sengkang E Wy, Singapore 544886, Singapore; Department of Cardiology, National Heart Centre Singapore, 5 Hospital Dr, Singapore 169609, Singapore; Department of Endocrinology, Sengkang General Hospital, 110 Sengkang E Wy, Singapore 544886, Singapore

**Keywords:** Phaeochromocytoma, Phaeochromocytoma multisystem crisis, Catecholamines, Case report

## Abstract

**Background:**

Phaeochromocytoma multisystem crisis (PMC) is characterized by labile blood pressures (extremes of hypo- and/or hypertension) and multiorgan failure as a result of catecholamine excess. Initial stabilization requires pharmacological and/or mechanical circulatory support, followed by the institution of antihypertensives to correct the underlying pathophysiology.

**Case summary:**

A previously well 40-year-old male developed a sudden onset of breathlessness. On presentation, he was in shock with multiorgan failure. He required intubation, mechanical ventilation, dual inotropic support, and renal replacement therapy. Bedside echocardiogram showed a severely impaired left ventricular ejection fraction (LVEF) of 25%. Coronary angiography revealed normal coronary arteries. In view of raised inflammatory markers and transaminitis, a computed tomography abdomen/pelvis was performed. An incidental left adrenal mass was found. Further work-ups revealed raised plasma metanephrine and normetanephrine, 24-h urine epinephrine, and norepinephrine. A cardiac magnetic resonance (CMR) showed myocardial inflammation and reverse Takotsubo pattern of regional wall motion abnormality (RWMA). The diagnosis of cardiogenic shock and stress cardiomyopathy secondary to PMC was made. He was subsequently initiated on α- and β-blockers and goal-directed medical therapy for heart failure. A ^68^Ga-DOTATATE scan showed avid tracer uptake of the left phaeochromocytoma. An interval CMR 3 weeks from presentation showed near normalization of the LVEF and RWMA. He underwent a successful laparoscopic left adrenalectomy and was antihypertensive-free since.

**Discussion:**

The clinical suspicion for PMC as the cause of cardiogenic shock requires astute clinical judgement, while the management requires an understanding of the underlying pathophysiology that calls for multidisciplinary inputs.

## Graphical abstract

**Graphical abstract:**
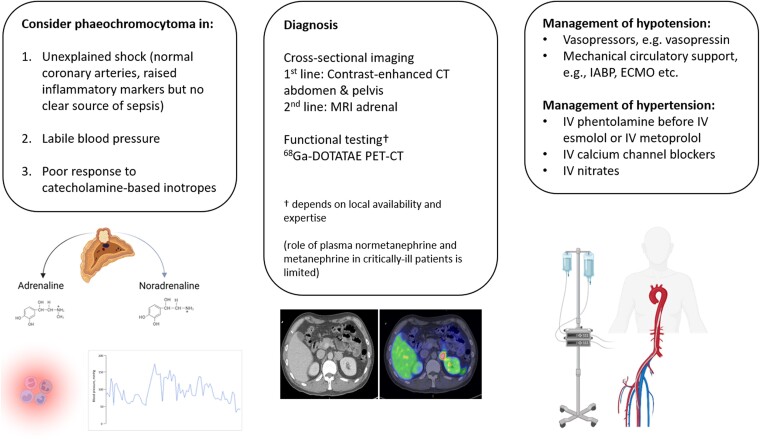

**Learning points**

- To entertain phaeochromocytoma multisystem crisis (PMC) as a cause of cardiogenic shock in:
  1. Unexplained shock state with no localizable source of sepsis.
  2. Labile blood pressure, i.e. severe hypo- and/or hypertension.
  3. Poor response to catecholamine-based inotropes, e.g. adrenaline, noradrenaline, dobutamine, etc.
- To recognize the strengths and limitations of conventional biochemistry and tomographic imaging at diagnosing PMC.
- Learn to navigate competing treatment priorities and work collaboratively with other specialities.

## Introduction

Phaeochromocytoma multisystem crisis (PMC) is a rare, but potentially fatal presentation of catecholamine overproduction, manifesting as a tetrad of multiorgan failure, high fever, encephalopathy, and blood pressure lability, i.e. extremes of hypo- and/or hypertension.^1^ First described in 1988 by Newall *et al.*, PMC differs from catecholamine-induced hypertensive crisis as hypertension may or may not be present in PMC initially.^1^ Phaeochromocytoma multisystem crisis is exceedingly rare, accounting for 19% of phaeochromocytoma crises reported in the literature.^2^ The actual number could be smaller after adjusting for publication bias. The prevalence of PMC among cardiogenic shock patients is unknown. This may be due to the rarity of the disease, lack of uniform diagnostic criteria, and under-recognition among the cardiology community.

Phaeochromocytoma is a great masquerader that can present to the cardiology service with arrhythmias, acute coronary syndrome, cardiomyopathy, or cardiogenic shock.^3,4^ The incidence and clinical presentation of phaeochromocytoma is an ever-changing landscape.^5^ A systematic review comparing studies before 2000 to those after 2010 has shown a three-fold increase in the incidence of phaeochromocytomas with the wider adoption of tomographic imaging and genetic screening.^6^ Most patients with phaeochromocytomas were asymptomatic at diagnosis. The classical triad of paroxysmal headache, palpitation, and sweating was only present in <25% of patients with phaeochromocytomas.^5^

Cardiogenic shock caused by the PMC crisis is a unique state of sudden catecholamine overproduction. The initial stabilization, diagnosis, choice of antihypertensives, timing of antihypertensive initiation, and surgical planning in patients with PMC and cardiogenic shock require risk-benefit evaluation from multidisciplinary stakeholders.

## Case presentation

A 40-year-old Chinese male with no significant past medical history presented with acute respiratory distress. He was previously well with no antecedent symptoms until the day of the presentation. In particular, there were no palpitations, diaphoresis, weight loss, or medication use. There was no recent travel history or viral illness. Earlier in the day, he drank two pints of beer, following which he had multiple episodes of vomiting and decided to seek medical attention.

**Figure 1:**
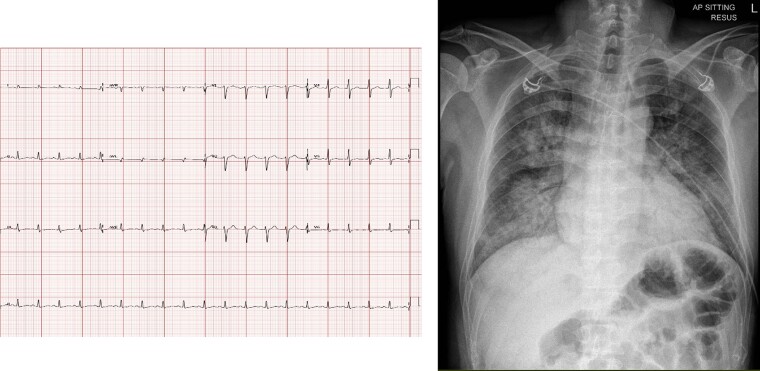
(Left) Initial electrocardiogram showing sinus tachycardia and no ischaemic changes. (Right) Chest X-ray on presentation, showing pulmonary congestion.

On arrival at the Emergency Department, he had a respiratory rate of 40 breaths/min, heart rate of 140 b.p.m., blood pressure of 79/61 mmHg, temperature of 37.5°C, and oxygen saturation of 80% on room air. Physical examination revealed bilateral lung crepitations and cold peripheries. The abdominal and neurological examination was unremarkable. The electrocardiogram on presentation showed sinus tachycardia with no ischaemic changes, and the chest X-ray showed pulmonary congestion (*Figure 1* and Supplementary material online, *Figure S1*). Given the respiratory distress and Type 1 respiratory failure, he was intubated. His blood pressure continued to drop, requiring dual inotropes, i.e. noradrenaline and dobutamine. A bedside echocardiogram showed severely impaired systolic function, a left ventricular ejection fraction (LVEF) of 25%, and regional wall motion abnormality (RWMA) (see Supplementary material online, *Videos S1–S4*). The initial troponin-T was 5744 ng/L (normal ≤29 ng/L), and NT-proBNP was 14 472 pg/mL (normal ≤99 pg/mL). Venous blood gas showed pH 7.240, BE −5.0 (normal −2.0–3.0), bicarbonate 22.7 mmol/L (normal 22.0–26.0) mmol/L, and the lactate was 9.6 mmol/L (0.5–2.2) mmol/L. An urgent coronary angiogram showed normal coronary arteries. Intra-aortic balloon pump (IABP) was inserted. He was initiated on continuous veno-venous hemofiltration in view of acute renal failure, oliguria, and severe acidosis.

**Figure 2:**
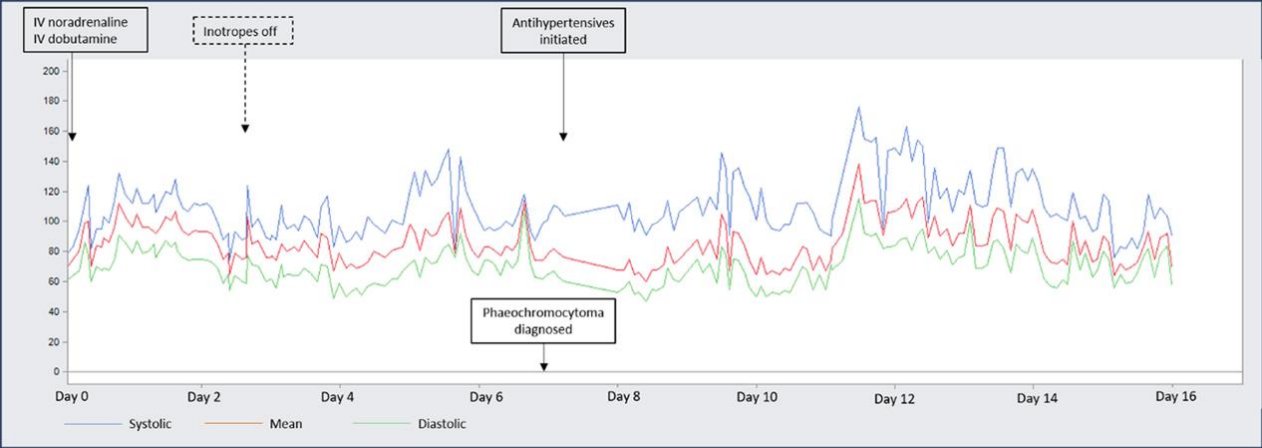
Cyclical arterial blood pressure spikes, at times reaching arterial blood pressure >130/80 mmHg despite severely impaired systolic function and poor cardiac output.

**Figure 3:**
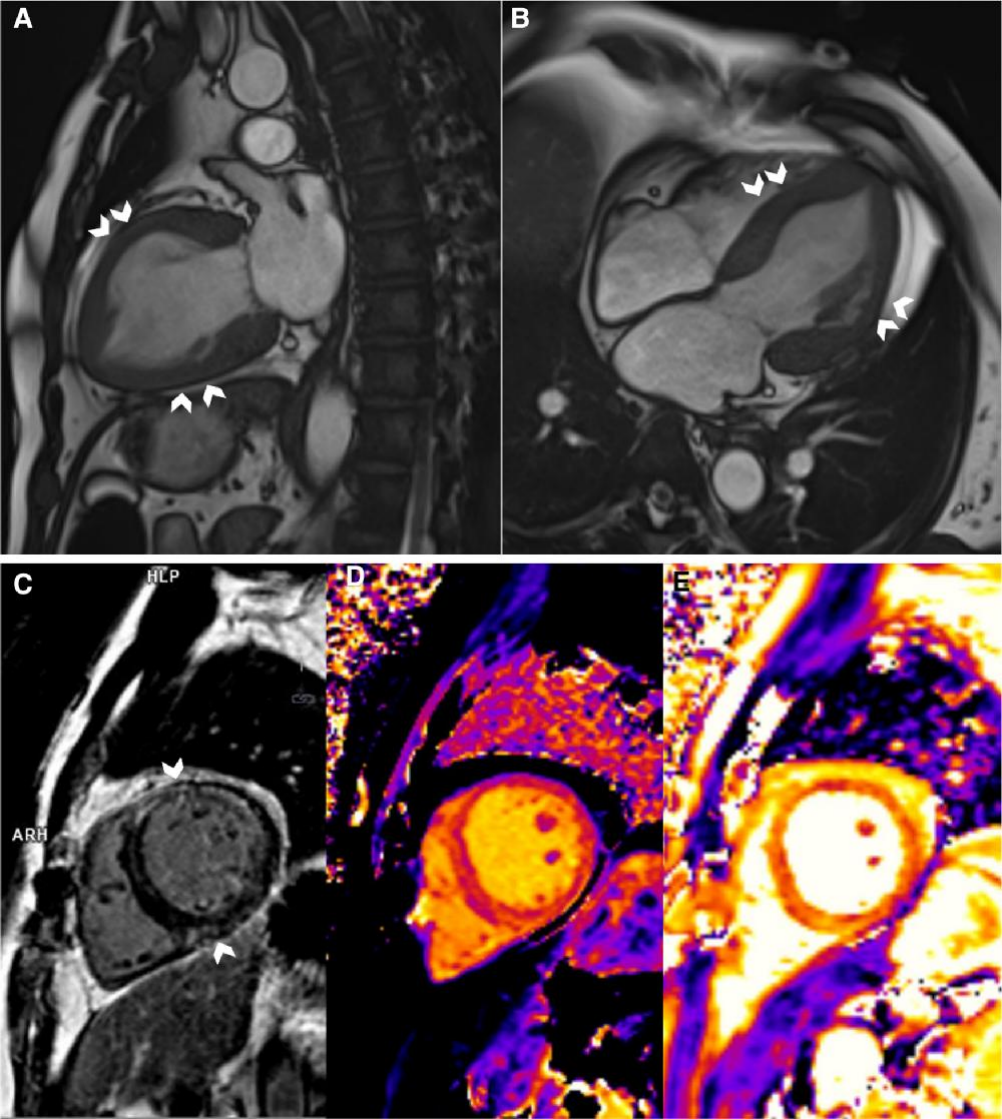
Cardiac magnetic resonance imaging. (*A* and *B*) Systolic frames of cine images showing akinesia of the mid-segments, i.e. mid-anterior, septal, lateral, and inferior (chevron arrows), and normokinesia of the basal and apical segments. (*C*) Mid-wall Late Gadolinium enhancement at the mid-anterior, anteroseptal, anterolateral, and inferior (chevron arrows) segments. (*D* and *E*) Parametric mapping of the corresponding slice, showing increased global native T1 (*D*) and T2 (*E*) relaxation times, implying the presence of oedema.

Despite being in extremis on presentation, he steadily recovered in the Intensive Care Unit. By Day 3, he was weaned off IABP and all inotropes. Cardiac magnetic resonance (CMR) showed a reduced LVEF of 35% and reversed the Takotsubo pattern of RWMA (*Figure 3*, Supplementary material online, *Video S5*). His inflammatory markers remained raised, total white 35.7 × 10^9^/L (normal 4.0–10.0 × 10^9^/L), C-reactive protein 146 mg/L (normal ≤4.9 mg/L). There was also acute liver injury with elevation in alanine transaminase 511 U/L (normal 6–66 U/L) and aspartate transaminase 456 U/L (normal 12–42 U/L). A computed tomography (CT) of the abdomen and pelvis was arranged to exclude intra-abdominal sepsis. No septic source was found but an incidental left adrenal mass was detected. The adrenal lesion was further characterized with a dedicated CT adrenal imaging protocol and found to be heterogenous, measuring 2.7 × 2.2 cm with attenuation values of 41 Hounsfield units (HUs) on the unenhanced phase, 81 HU on the portal venous phase and 66 HU on the delayed enhancement phase with an absolute washout of 41% and relative washout of 18%. As the lesion was deemed indeterminate, an Endocrinologist was consulted, and phaeochromocytoma was suspected based on the clinical presentation (*Figure 2*). Subsequent serum and urine biochemistry investigations revealed raised plasma-free metanephrine 2.98 nmol/L (normal <0.33 nmol/L) and normetanephrine 1.32 nmol/L (normal <0.71 nmol/L); elevated 24-h urine epinephrine 1603 nmol/day (normal 3–109 nmol/day), norepinephrine 653 nmol/day (normal 89–473 nmol/day).

**Figure 4:**
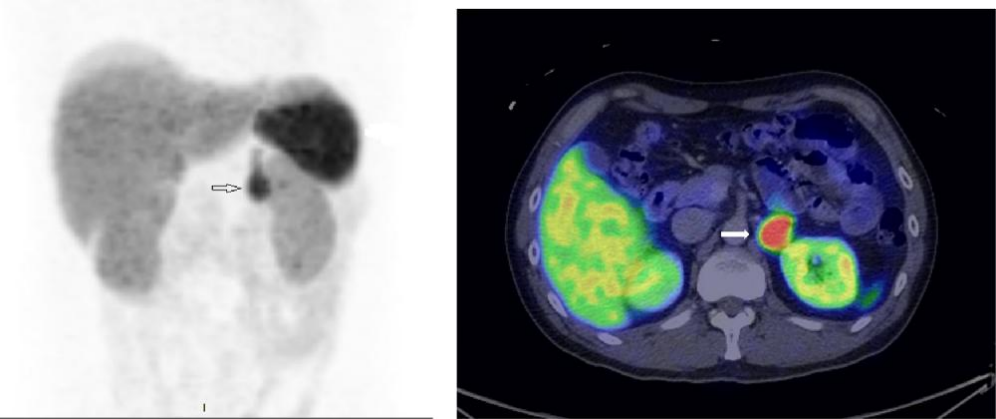
68Ga-68 DOTATATE scan showing avid tracer uptake by the left phaeochromocytoma (indicated by the arrows).

**Figure 5:**
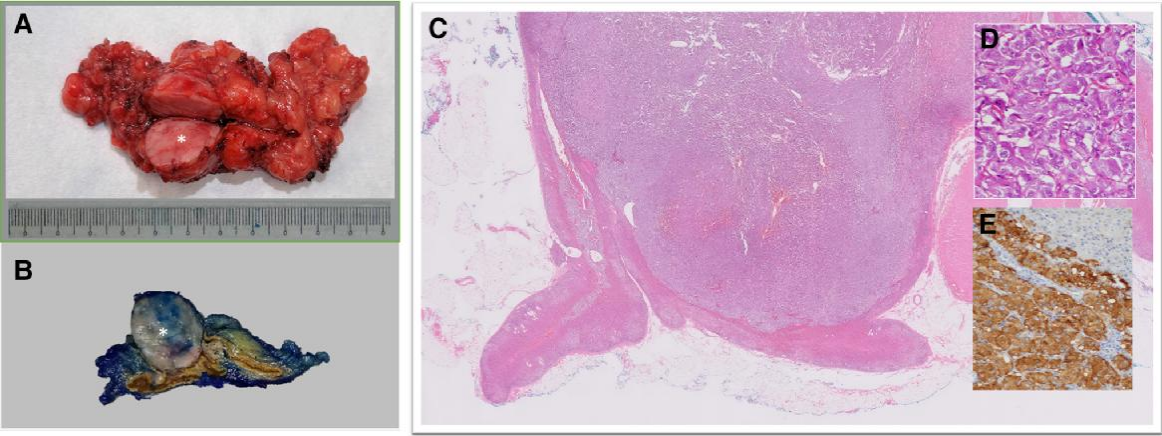
(*A*) Gross surgical specimen. Phaeochromocytoma (round, fleshy mass, asterisk) with adjacent normal adrenal structures and surrounding fat. (*B*) Phaeochromocytoma (well-circumscribed, cream-coloured mass, asterisk) arising from the medulla. The normal adrenal cortex appears dark yellow and the normal medulla appears brown. (*C*) Low power H&E specimen. Nodular mass (purple) involving the medulla of the adrenal gland. A small amount of normal adrenal gland is present (bright pink). (*D*) High-power H&E specimen. The tumour has a *Zellballen* (German for ball of cells) pattern, large and ovoid nuclei with nucleoli, and abundant granular cytoplasm. (*E*) Tumour expressing neuroendocrine markers, i.e. synaptophysin and chromogranin positive. Note that the normal adrenal cortex in the right upper corner is stained negative.

A treatment regimen of intravenous (IV) phentolamine and esmolol, as well as a high sodium diet (>5 g/day), was proposed. From the endocrinologist’s point of view, the IV infusion route provides rapid and titratable reversal of catecholamine-over stimulation. From the cardiologists’ point of view, starting IV antihypertensives in patients who were just weaned off dual inotropes and prescribing Sodium Chloride tablets to heart failure patients with severely impaired LVEF was not without trepidation. A compromise was reached that the patient was given oral α-blocker (phenoxybenzamine) gradually with haemodynamic monitoring in the high dependency unit (HDU) following which bisoprolol was initiated to keep the heart rate <80/min. The patient remained in the HDU for a further week and was transferred to the general ward. In the general ward, he had short runs of monomorphic ventricular tachycardia. He was treated conservatively with up-titration of β-blocker. He was discharged well on Day 23 with three oral antihypertensives, i.e. phenoxybenzamine, bisoprolol, amlodipine, and sodium chloride tablets. A CMR showed LVEF of 53%, improvement of the RWMA, and resolution of late Gadolinium enhancement despite the presence of oedema. An outpatient ^68^Ga-DOTATATE scan confirmed active phaeochromocytoma (*Figure 4*) without evidence of metastases or multifocality. Two months after the initial presentation, the patient underwent a successful elective laparoscopic left adrenalectomy by the urologist (*Figure 5*). The patient was off all antihypertensives and discharged well on post-operative Day 3.

## Discussion

We hereby present a case of cardiogenic shock with labile blood pressure, pulmonary oedema, acute renal insufficiency, and ischaemic hepatitis secondary to PMC. Although rare, it carries a high mortality rate, ranging from 28% to 85% in the literature.^7^ Thus, the authors propose that it is worthwhile considering PMC as a cause of cardiogenic shock in the following circumstances:

1. Unexplained shock state with raised inflammatory markers but no localizable source of sepsis and in the absence of significant coronary artery disease or valvular disease.
2. Labile blood pressure, i.e. severe hypo- and/or hypertension.
3. Poor response to catecholamine-based inotropic agents, e.g. adrenaline, noradrenaline, dobutamine, etc.

The pathophysiology of PMC is not well understood. Both adrenaline and noradrenaline-secreting tumours have been associated with phaeochromocytoma crisis.^7^ In fact, 73% of the reported phaeochromocytoma crises had a noradrenaline phenotype.^7^ Our patient has a predominantly adrenergic biochemical phenotype. Adrenaline-secreting tumours can cause excessive myocardial and vascular stimulation via its β_2_-adrenergic action, potentially triggering myocardial infarction or causing repressed myocardial contractility with stress cardiomyopathy or vasodilation with profound hypotension.^8^ Noradrenaline-secreting tumours cause excessive vasoconstriction via its α_1_-adrenergic action, resulting in coronary artery vasospasm, acute coronary syndrome, or pulmonary oedema.^8^ Although different catecholamines have different adrenoceptor affinities and, consequently, different clinical manifestations, studies have shown that the nature of phaeochromocytoma crisis was not influenced by the type of catecholamine secreted by the tumours.^7^

In our case, the left phaeochromocytoma was discovered incidentally on CT abdomen. The patient was previously asymptomatic and normotensive. On the day of the presentation, he drank beer, which contains tyramine that could enhance catecholamine release and had been associated with phaeochromocytoma crisis. The mechanisms that precipitate phaeochromocytoma crisis are not well understood. A few precipitants have been associated with phaeochromocytoma crisis, although their causality cannot be implicated due to the observational nature of the data. Examples of these precipitants include, but are not limited to that of tumour haemorrhage or infarction, surgical manipulation of the tumour, abdominal trauma, general anaesthesia, and drugs e.g. steroids, glucagon, tricyclic antidepressants, sympathomimetics, and β-blockers (before adequate α-blockade).^9^

In this case, the initial diagnosis of phaeochromocytoma was not without challenges. The interpretation of biochemistry, i.e. plasma and urinary metanephrines, i.e. peripherally converted catecholamine metabolites, was confounded by a few factors that are known to cause false positives. There was recent use of catecholamine-based inotropes, which ideally should be stopped at least 2 weeks before testing. Besides, critical illness and physical or psychological stress have been known to cause elevated metanephrines.^8^ Measurements of plasma-free or urinary fractionated metanephrines as recommended by current guidelines as initial diagnostic tests may not apply during pheochromocytoma crises. While these tests are highly sensitive (97%–99%), they are relatively less specific (69%–89%),^10^ rendering them fraught with high false positive rates and less suitable in the emergency setting due to confounders.^11^ In acute settings, anatomical imaging, e.g. CT or magnetic resonance imaging (MRI) of the adrenal is usually preferred over biochemical testing.^11^ CT adrenal is usually the first-line imaging modality due to its availability and high spatial resolution. It has a sensitivity of 89%–95% in diagnosing phaeochromocytomas. Generally, but not always, phaeochromocytomas tend to be large, i.e. >3 cm, heterogenous and enhance avidly, i.e. >130 HUs on post-contrast CT.^12^ Nuclear imaging can be considered in case of equivocal or negative CT/MRI. A functional study, radionuclide imaging has the added value of locating extra-adrenal tumour and/or metastasis. Recent studies have shown good sensitivity of somatostatin receptor-based PET/CT, e.g. ^68^Ga-DOTATATE PET/CT at detecting both primary and metastatic phaeochromocytoma.^12^ Imaging strategy should be individualized, based on local expertise and availability, considering tumour heterogeneity, i.e. genotype and biochemical phenotype, expected tumour location or size, and patient factors, e.g. age and preference.^12^

There is currently no consensus on the treatment of hypotension in PMC. While the use of catecholamine-based inotropes, e.g. adrenaline, noradrenaline, and dopamine have been described in the literature,^13^ their mechanism of action in a state of catecholamine excess is unclear. Although the use of vasopressin has been described,^14^ whether it should be preferred over catecholamine-based inotropic agents needs further studies. The role of fluid resuscitation is equally uncertain, especially in the presence of impaired LV systolic function. Theoretically, hypotension in phaeochromocytoma crisis is often fluid-responsive as fluid corrects the intravascular volume depletion from chronic α_1_-adrenoceptor-mediated vasoconstriction.^8^ However, hypotension in phaeochromocytoma crisis can also be caused by excessive β_2_-adrenoceptor-mediated myocardial ischaemia or stress cardiomyopathy, in which case fluid resuscitation, if ever indicated, should be administered with caution. In such a scenario, the early use of adjunctive mechanical circulatory support, e.g. IABP and extracorporeal membrane oxygenation in PMC, should be considered during the initial stabilization.^7,8,13^ A pooled analysis of published cases has shown better survival in patients who received mechanical circulatory support compared to those who were managed conservatively.^7^

While α-blockade is usually the conventional medical treatment in patients with phaeochromocytoma-induced hypertensive crisis, its use in patients with PMC is limited, if not contraindicated, due to the presence of profound hypotension. In catecholamine-induced hypertensive crisis, α-blocker addresses the underlying pathophysiology by reversing the vasoconstriction and resultant hypertension. It is usually administered intravenously to provide rapid and titratable blockade. Intravenous phentolamine is the first-line α-blocker due to its fast onset and short duration of action. In active or recovering PMC, the timing of α-blocker initiation, the route of administration, and the choice and dose of medication are matters of expert opinion. In our case, an oral non-selective and non-competitive α-blocker (phenoxybenzamine) was initiated once the patient was stabilized, i.e. weaned off inotropes and IABP. While studies have shown that α-blockade was strongly associated with survival in patients with phaeochromocytoma crisis,^7^ It is worth noting that survival bias could be at play. Patients in whom α-blocker could be started, i.e. not hypotensive, were self-selected to survive.

β-Blocker, a cornerstone therapy in heart failure with reduced ejection fraction, if required, is usually initiated after adequate α-blockade. The use of β-blockers before adequate α-blockade has been associated with hypertensive crisis and, in some cases, acute heart failure. Theoretically, blocking the β-receptors that promote vasodilation could cause unopposed α-adrenergic vasoconstriction. In a catecholamine-induced hypertensive crisis, β1-selective blockers, e.g. IV esmolol and IV metoprolol, are preferred over non-selective blockers, e.g. IV propranolol.^8,15^

A high-sodium diet (3–5 g/day) is recommended following initiation of α-blocker before elective adrenalectomy.^10^ Retrospective studies have shown that a high-sodium diet reverses catecholamine-induced blood volume contraction, prevents orthostatic hypotension, and avoids significant post-operative hypotension.^16^ However, its role in PMC is unclear, especially in the presence of acute decompensated heart failure and renal failure.

Due to its rarity and patient accrual challenges, PMC remains poorly understood, requiring further research. It is unlikely that the management of PMC would be guided by large, prospective study, let alone randomized controlled trial. In the absence of such evidence, management of PMC should be guided by an understanding of the underlying pathophysiology. Indeed, many of the PMC treatment strategies are extrapolated from principles of hypertension management in patients with catecholamine-induced hypertensive crisis or haemodynamically stable pre-operative patients undergoing elective adrenalectomy.

Phaeochromocytoma multisystem crisis is an exceedingly rare, but potentially lethal cause of cardiogenic shock. The diagnosis is elusive and often missed. Early recognition of secondary causes of cardiogenic shock, in this case, an extreme manifestation of a rare neuroendocrine tumour, requires astute clinical judgment. Patients with cardiogenic shock secondary to PMC should be comanaged by cardiologists and endocrinologists, each with a good understanding of the other’s domain knowledge and both willing to work through, at times, competing treatment priorities.

## Supplementary Material

ytae463_Supplementary_Data

## Data Availability

The data underlying this article are available in the article and its online Supplementary material.

## Lead author biography

Dr Go is a cardiologist with special interest in multimodality imaging and valvular heart disease.
