# Supplementary figures and images for: Cardiogenic shock in phaeochromocytoma multisystem crisis: a case report
Source: Eur Heart J Case Rep. 2024 Sep 9;8(9):ytae463. doi: 10.1093/ehjcr/ytae463 (PMC11420679; doi:10.1093/ehjcr/ytae463)

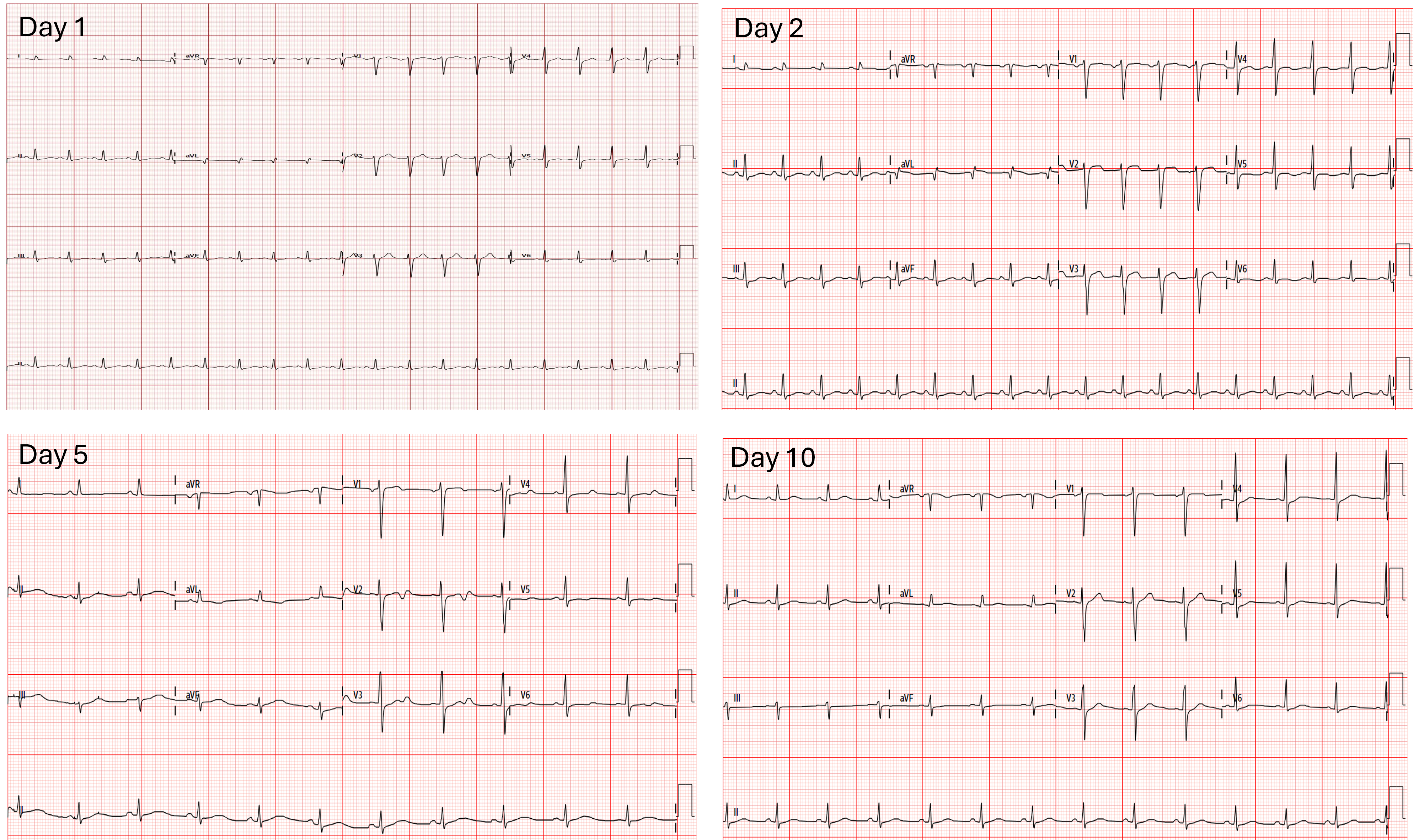

Supplement: ytae463_Supplementary_Data [file ytae463_supplementary_data.zip › Serial ECGs.png]
